# Effects of UV-B Radiation on the Performance, Antioxidant Response and Protective Compounds of Hazelnut Pollen

**DOI:** 10.3390/plants11192574

**Published:** 2022-09-29

**Authors:** Aslıhan Çetinbaş-Genç, Orçun Toksöz, Chiara Piccini, Özkan Kilin, Nüzhet Cenk Sesal, Giampiero Cai

**Affiliations:** 1 Department of Biology, Faculty of Science, Marmara University, Kadıköy, Istanbul 34722, Turkey; 2 Institute of Pure and Applied Sciences, Marmara University, Istanbul 34722, Turkey; 3 Department of Life Sciences, University of Siena, Via Mattioli 4, 53100 Siena, Italy

**Keywords:** antioxidant activity, hazelnut, pollen, UV-B-absorbing compounds, UV-B radiation

## Abstract

Increasing ultraviolet (UV) radiation is expected to become a problem in hazelnut cultivation. The aim of this study is to examine the effects of UV-B on hazelnut pollen. To this end, the pollens were exposed to UV-B for 1, 2, and 3 h at distances of 10, 20, 30, and 40 cm. Groups treated for 2 h at 20 cm and 3 h at 10 and 20 cm were identified as the most affected based on the results of viability, germination, and tube elongation. Further studies on these groups showed that UV-B does not change the DPPH radical scavenging activity for all groups. However, total phenolic compounds decreased after 3 h of treatment at 10 and 20 cm, while total flavonoid compounds decreased after all treatment groups. The UV-B absorbance of cytoplasmic and cell-wall-bound fractions decreased for all groups. The UV-B absorbance of the sporopollenin-derived fraction increased after 2 h of treatment at 20 cm but decreases after treatment for 3 h at 10 and 20 cm. In summary, exposure to UV-B for different times and distances adversely affected pollen grains in terms of pollen viability, germination rate, tube length, and the level of antioxidant molecules and UV-absorbing compounds.

## 1. Introduction

Hazelnut (*Corylus avellana*) is an economically important plant belonging to the Betulaceae family [1]. It is generally grown in the northern hemisphere of the world; Turkey, Italy and Azerbaijan are the main producers of hazelnuts [2]. The demand for hazelnuts in the chocolate and confectionery industry, the main users of hazelnuts, is increasing progressively [3]. Parallel to the increase in demand, studies to improve the production of hazelnuts with the aim of investigating in depth the factors that influence the product yield are becoming increasingly demanding [4]. In addition to these, due to easier access to raw materials, studies to improve the cultivation of hazelnut in different ecological contexts and to increase the cultivation of hazelnut in the southern hemisphere are constantly increasing [5]. All this indicates that hazelnut cultivation will soon be widespread in regions with different ecological characteristics. The most important factor influencing crop yield and quality in plants is probably environmental stresses caused by climate change; therefore, it is very important and realistic to focus on the effects of environmental stressors on the vegetative and generative development of hazelnut in studies conducted to increase yield [6]. From the point of view of reproductive biology, the most important factor affecting the yield of hazelnut is delayed fertilization [7]. Since the ovary is not sufficiently differentiated at the time of pollination, pollen grains must wait for the ovary to mature for about 2–3 months [8,9]. During this period, the success of pollination and fertilization is at risk since pollen grains can lose their efficiency by being exposed to various environmental stresses, such as temperature, drought, salinity, air pollutants and ultraviolet radiation [10]. Among these stressors, UV radiation is a little-studied stressor for pollen grains [11]. However, given the progressive depletion of the ozone layer, it is expected that UV radiation will become a common problem capable of influencing yield both in existing hazelnut-growing regions and in other ecological areas where cultivation has increased. Because of all this, it is clearly important to understand the effects of UV radiation on hazelnut pollen at different levels. Another reason is that the amount of information available in the scientific literature is still limited and does not provide a clear view of the effect of this environmental stressor on the pollination biology of hazelnut.

UV radiation is classified according to the different wavelengths: UV-C (100–280 nm), UV-B (280–315 nm) and UV-A (315–400 nm) [12]. UV-B is the most dangerous part of UV radiation and can induce a variety of harmful effects in living organisms [13]. In addition, stratospheric ozone dynamics and climate change gradually increase the harmful effects of UV-B radiation on organisms [14]. Plants are invariably exposed to UV-B radiation, as they need exposure to light for photosynthetic activity [15]. However, it is known that exposure to UV-B radiation can limit the growth and development of plants by directly or indirectly causing various adverse effects [16,17]. Most studies have focused mainly on the vegetative development of plants while the effects of UV-B on plant reproductive development have not received much attention [15,18,19], whereas this is even more important because plant reproduction and reproductive structures are vulnerable to UV-B radiation [15]. Specifically, pollen grains are the reproductive structures of plants that are less protected, therefore more exposed, and much more sensitive to UV-B radiation [20,21]. In addition, UV-B-induced morpho-physiological changes in pollen grains can also cause economic losses, as they affect the yield and quality of the product [15]. This underlines the need for further studies on the effects of UV-B radiation at the level of plant reproductive structures, particularly on pollen. This is also corroborated by studies reporting that UV-B induces decreased pollen viability, pollen germination and tube length in crop species such as soybeans, olives, and maize [22,23,24].

The main parameters used to assess the effects of environmental stresses on pollen are pollen viability and pollen germination rate [25]. Pollen grains with high viability and high germination capacity are necessary for fertilization because non-viable pollen grains cannot form pollen tubes and cannot transfer sperm nuclei to the embryo sac for fertilization [26]. In addition, for fertilization to take place, the pollen tubes must be long enough to reach the embryo sac by growing along the style; therefore, another key parameter for assessing the effects of environmental stressors on pollen is the length of the pollen tube [27]. To extend the basic assessments, it is also essential to focus on antioxidant activity and changes in phenolic and flavonoid content; this is because excessive stress-induced ROS production (which limits pollen germination capacity and tube elongation) must be removed by enzymatic antioxidants such as superoxide dismutase (SOD), catalase (CAT) and peroxidase [20,28,29]. In addition, phenolic compounds also show antioxidant properties that protect cells and tissues from the toxic effect of ROS [30]. Flavonoids are the most effective phenolic compounds in the response to environmental stresses such as UV because they effectively absorb UV-B radiation and strongly neutralize ROS [31]. Moreover, under exposure to excessive light or UV-B radiation, the synthesis of more effective flavonoids (with dihydroxy B ring substitute) increases rapidly compared to others less effective [24]. Overall, these can be called UV-B-absorbing compounds (UACs) because they protect pollen grains from oxidative stress by absorbing UV-B and because of their antioxidant capacity [32]. Pollen UACs are found both in the soluble cytoplasmic fraction, in the cell-wall-bound insoluble fraction, and in the sporopollenin-derived insoluble fraction [33,34]. UACs (especially in the sporopollenin-derived fraction) respond to UV radiation by modifying their phenolic compounds in combination with exposure to UV radiation [35]. Therefore, pollen UACs may be a suitable indicator for monitoring the effects of UV radiation on pollen grains [36,37]. Moreover, the exine layer of pollen is composed of sporopollenin, which is an extremely robust mix of biopolymers resistant to physical, biological, and chemical degradation procedures [34,35]. Sporopollenin responds to UV radiation by changing its phenolic compounds in conjunction with exposure to UV radiation [35]. Therefore, exine can be used as a suitable indicator to monitor the effects of UV radiation on pollen grains [34].

The aim of this study is to examine the effects of constant and intense UV-B radiation on hazelnut pollen, which is expected to occur both in current cultivated areas and in areas where cultivation is increasing. To this end, the effects of UV-B radiation on hazelnut pollen grains were examined by focusing on pollen viability, germination rate and pollen tube length, as well as alterations in antioxidant activity, the level of phenols, flavonoids, and UACs.

## 2. Results

**Figure 1:**
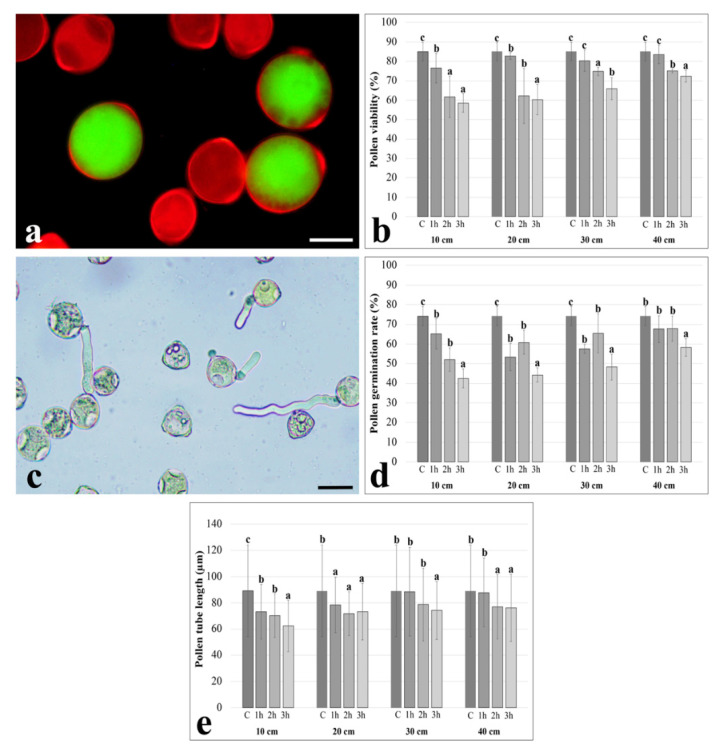
The effects of UV-B radiation on the main parameters of pollen grains. (**a**) Representative images of FDA/PI staining providing green fluorescence radiation for viable pollen grains and red fluorescence radiation for non-viable pollen grains; (**b**) Pollen viability rates (%) after 1, 2, and 3 h treatment at 10, 20, 30, and 40 cm distances; (**c**) Representative images of germinated pollen grains and pollen tubes; (**d**) Pollen germination rates (%) after 1, 2, and 3 h treatment at 10, 20, 30, and 40 cm distances; (**e**) Pollen tube lengths (µm) after 1, 2, and 3 h treatment at 10, 20, 30, and 40 cm distances. Different letters within the figures indicate statistically significant differences. Bar: 20 µm.

Initially, we measured pollen viability rates, germination rates and pollen tube lengths to provide information on the effects caused by UV-B radiation on the main functional parameters of pollen. According to data obtained from FDA/PI staining (which provides green fluorescence for viable pollen grains and red fluorescence for non-viable pollen grains) (Figure 1a), all treatment times significantly reduced pollen viability compared to the control in the case of pollen grains exposed to 10 and 20 cm. However, no significant difference was observed between 2 and 3 h of treatment at 10 cm and between 1 and 2 h of treatment at 20 cm. Pollen viability rates were significantly reduced compared to the control only after UV-B irradiation for 2 and 3 h in the case of pollen grains exposed 30 and 40 cm away (Figure 1b).

Next, we evaluated pollen germination (Figure 1c). Germination rates decreased significantly compared to the control after each of treatment time, 10, 20 and 30 cm away from the lamp. Although no significant difference was observed between 1 and 2 h of treatment at 10, 20 and 30 cm, pollen germination rates were significantly reduced compared to the control only after 3-hour UV-B irradiation in the case of pollen grains exposed at 40 cm (Figure 1d). Pollen tube lengths were significantly reduced compared to the control for all treatment times at distances of 10 and 20 cm. However, there was no significant difference between 1 and 2 h of treatment at 10 cm and between 1, 2 and 3 h at 20 cm. The lengths of pollen tubes were significantly reduced compared to the control only after 3 h to 30 cm and only after 2 and 3 h at 40 cm. However, there was no significant difference between the 2 and 3 h of treatment at 40 cm (Figure 1e).

To identify the experimental conditions showing the highest sensitivity on pollen grains, the CSRI value of all groups was calculated. Based on cumulative stress response index (CSRI) data, pollen grains exposed to UV-B rays for 1 h at 40 cm were not affected. Pollen grains showed tolerance to UV-B exposure for 1 h of treatment at 10, 20, and 30 cm and for 2 h oh of treatment at 30 and 40 cm. Pollen grains were moderately affected by UV-B exposure after 2 h of treatment at 10 cm and a 3 h treatment at 30 and 40 cm. The most acute effects caused by UV-B exposure were found after 2 h of treatment at 20 cm, 3 h of treatment at 10 cm and 3 h of treatment at 20 cm, respectively (Table 1). Based on these early results, we have identified three main groups that show the most acute effects, as evidenced by CSRI values. The most stressful conditions were 2 h at 20 cm and 3 h of treatment at 10 and 20 cm. As we aimed to reveal the damaging effects of UV on pollen grains, we then carried out additional studies (DPPH%, total phenolic compounds, total flavonoid compounds and UV-B absorbance of cytoplasmic fractions, cell-wall-bound fractions and sporopollenin-derived fractions) on those groups showing more acute effects caused by exposure to UV-B rays.

**Figure 2:**
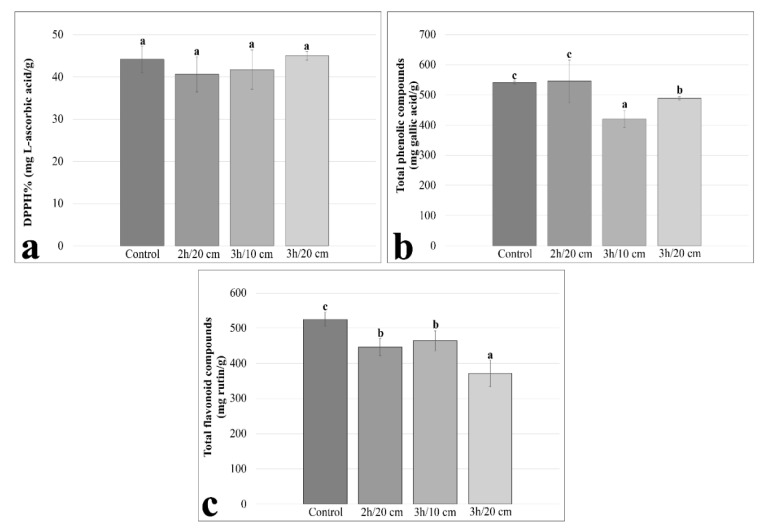
The effects of UV-B radiation on antioxidant activity, total phenolic compounds, and total flavonoid compounds on the groups showing more acute effects caused by exposure to UV-B rays. (**a**) DPPH% activity after 2 h of treatment at 20 cm, 3 h of treatment at 10 cm and 3 h of treatment at 20 cm; (**b**); Total phenolic compounds after 2 h of treatment at 20 cm, 3 h of treatment at 10 cm and 3 h of treatment at 20 cm; (**c**) Total flavonoid compounds after 2 h of treatment at 20 cm, 3 h of treatment at 10 cm and 3 h of treatment at 20 cm. Different letters within the figures indicate statistically significant differences.

To monitor the effects caused by UV-B radiation on the antioxidant activities of pollen grains, we used the DPPH radical scavenging test. According to our results, UV-B radiation did not cause any significant change in DPPH activity after treatments (Figure 2a). To assess the antioxidant response, we also measured the total phenolic content. Total phenolic compounds significantly decreased compared to the control by 22% after a 3 h treatment at 10 cm and by 9% after a 3 h treatment at 20 cm (Figure 2b). In addition to the total phenolic content, we also measured flavonoid content as an antioxidant response index. Total flavonoid compounds decreased significantly compared to the control after all treatments and at all distances. Total flavonoid compounds decreased by 15% after 2 h of treatment at 20 cm, by 11% after 3 h of treatment at 10 cm and by 29% after 3 h of treatment at 20 cm. There was no significant difference between the 2 h of treatment at 10 cm and the 3 h treatment at 10 cm (Figure 2c).

**Figure 3:**
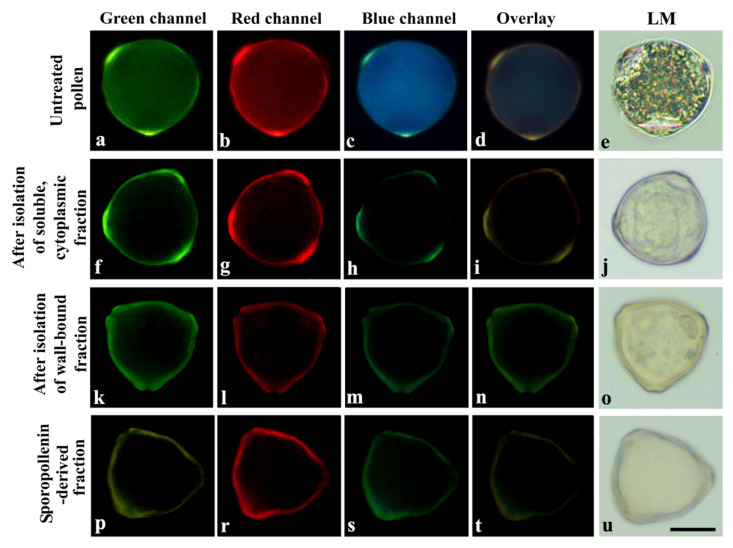
Representative fluorescence microscope images of pollen grains before and the after the isolation protocols. (**a**) Micrograph of untreated pollen under green channel; (**b**) Under red channel; (**c**) Under blue channel; (**d**) Under overlay of green, red and blue channel; (**e**) Under light microscope; (**f**) Micrograph of pollen after isolation of soluble, cytoplasmic fraction under green channel; (**g**) Under red channel; (**h**) Under blue channel; (**i**) Under overlay of green, red and blue channel; (**j**) Under light microscope; (**k**) Micrograph of pollen after isolation of wall-bound fraction under green channel; (**l**) Under red channel; (**m**) Under blue channel; (**n**) Under overlay of green, red and blue channel; (**o**) Under light microscope; (**p**) Pollen of sporopollenin-derived fraction under green channel; (**r**) Under red channel; (**s**) Under blue channel; (**t**) Under overlay of green, red and blue channel; (**u**) Under light microscope. Bar: 10 µm.

To examine the effects of UV-B radiation on UACs in pollen grains, we calculated the UV-B absorbance values of UACs in the three different pollen fractions (cytoplasmic fraction, cell-wall-bound fraction and sporopollenin-derived fraction) obtained by sequential extraction. First, we examined pollen grains under a fluorescence microscope to determine if the extraction process was successful. Based on our results, untreated pollen grains showed strong fluorescence due to the high content of internal components capable of absorbing UV-B (Figure 3a–d). The cytoplasmic content was also very noticeable under the light microscope (Figure 3e). After isolation of the cytoplasmic fraction, a decrease in autofluorescence in the pollen cytoplasm was observed (Figure 3f–i), while observations under the light microscope confirmed the removal of pollen cytoplasm (Figure 3j). After isolation of the cell-wall-bound fraction, a decrease in cell wall autofluorescence was found (Figure 3k–o). The autofluorescence of the sporopollenin exine capsule obtained at the end of the isolation protocols was quite evident (Figure 3p–u).

**Figure 4:**
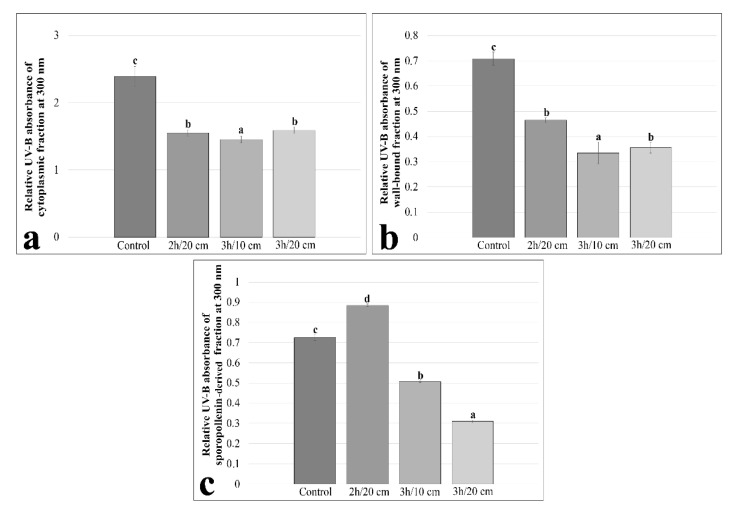
The effects of UV-B radiation on relative UV-B absorbance of different fractions on the groups showing more acute effects caused by exposure to UV-B rays. (**a**) UV-B absorbance of cytoplasmic fraction after 2 h of treatment at 20 cm, 3 h of treatment at 10 cm and 3 h of treatment at 20 cm; (**b**) UV-B absorbance of wall-bound fraction after 2 h of treatment at 20 cm, 3 h of treatment at 10 cm and 3 h of treatment at 20 cm; (**c**) UV-B absorbance of sporopollenin-derived fraction after 2 h of treatment at 20 cm, 3 h of treatment at 10 cm and 3 h of treatment at 20 cm. Different letters within the figures indicate statistically significant differences.

We measured UV-B absorbance of cytoplasmic fractions, cell-wall-bound fractions and sporopollenin-derived fractions at 300 nm for all distances. For the cytoplasmic fraction, UV-B absorbance decreased significantly compared to the control for all analysis times and for all distances. UV-B absorbance decreased by 35% after 2 h of treatment at 20 cm, by 39% after a 3-hour treatment at 10 cm, and by 33% after a 3-hour treatment at 20 cm. However, there was no significant difference between the 2 h of treatment at 20 cm and the 3-hour treatment at 20 cm (Figure 4a). For the cell-wall-bound fraction, UV-B absorbance decreased compared to the control for all treatment times and for all distances. UV-B absorbance decreased by 34% after treatment for 2 h at 20 cm, by 52% after treatment for 3 h at 10 cm and by 49% after treatment for 3 h at 20 cm. However, there was no significant difference between the 2 h of treatment at 20 cm and the 3-hour treatment at 20 cm (Figure 4b). In the case of the sporopollenin-derived fraction, UV-B absorbance increased significantly by 22% compared to the control after 2 h of treatment at 20 cm. However, UV-B absorbance decreased significantly compared to the control by 30% after 3 h of treatment at 10 cm and by 57% after a 3-hour treatment at 20 cm (Figure 4c).

## 3. Discussion

The increase in UV-B radiation on earth due to ozone depletion causes multiple harmful effects on the vegetative and generative structures of plants [13]. In terms of generative structures, pollen grains are known to be more sensitive to environmental stresses, including UV-B radiation, than other generative tissues [34]. Therefore, there are many studies on the effects of different environmental stresses, including UV-B radiation, on pollen. For example, UV-B radiation has been found to reduce pollen viability in *Brassica rapa* and *Zea mays* [18,38]. In addition, UV-B radiation has been shown to inhibit pollen germination and pollen tube elongation in *Cleome lutea*, *Scrophularia peregrina* and *Geranium viscosissimum* [39], *Brassica rapa* [18], *Zea mays* [40,41], *Glycine max* [22], and *Paulownia tomentosa* [42]. Torabinejad et al. [43] examined the effects of UV-B radiation on pollen in 34 taxa and found that the germination rate in 5 taxa and the tube length in 19 taxa decreased due to UV-B radiation [43]. In addition, Feng et al. [20] examined the effects of UV-B radiation on pollen of 19 taxa and found that UV-B radiation reduced pollen germination rate and tube length. Koubouris et al. [23], by examining the effects of UV-B on olive pollen, found that UV-B radiation reduced pollen germination rate and pollen tube elongation. Not all UV-B effects are negative. For example, Chesnokov and Manteuffel [44] reported that low-dose UV-B radiation stimulated pollen tube growth in *Nicotiana plumbaginifolia*. Despite some experimental reports in which UV-B radiation stimulates pollen tube growth, these examples clearly show how sensitive pollen grains are to UV-B radiation. Like the experimental cases described above, UV-B radiation reduced the viability and germination rate of pollen and the elongation of the pollen tube in the hazelnut. According to CSRI values, the harmful effects of UV-B radiation on pollen grains are proportional to the exposure time but inversely proportional to the distance from the UV-B source. Still, according to the CSRI results, the most acute effects caused by exposure to UV-B radiation occur after 2 h of treatment at 20 cm, 3 h of treatment at 10 cm and 3 h of treatment at 20 cm, respectively.

It is known that when cells are exposed to UV-B radiation, the production of ROS may increase; however, the cells are able to reduce the intense accumulation of ROS by their antioxidant activity [20]. For this reason, UV-B-induced changes in antioxidant activity could provide insight into the effects of stress. Nevertheless, there are few pieces information describing the effects of UV-B on the antioxidant activities of pollen grains. For example, He et al. [45] reported that hydrogen peroxide (H_2_O_2_) is involved in the UV-B inhibition of pollen germination in vitro and pollen tube growth in *Paeonia suffruticosa* and *Paulownia tomentosa*. In addition, the inhibitory effects of UV-B radiation on pollen germination and pollen tube growth have been shown to reverse when UV-B-induced H_2_O_2_ is removed by the addition of ascorbate and CAT. Wang et al. [21] showed that UV-B decreases the scavenging activity of SOD, CAT, POD, and DPPH radicals in maize pollen; this indicates that UV-B radiation impairs the antioxidant capacity of pollen. In addition, phenolic compounds work as indicators of stress because they accumulate at high levels in many plant tissues in response to a wide range of abiotic signals [46]. For example, a decrease in phenolic compounds induced by heat stress has been reported in pollen grains of several citrus species [47]. Among phenolic compounds, flavonoids are the key molecules in the stress response [24]. Rezanejad [48] reported that air pollution induces the accumulation of flavonoids in pollen of *Spartium junceum*, *Lagerstroemia indica*, *Thuja orientalis* and *Petunia hybrida*. In addition to a decrease in phenolic compounds, heat stress also induced a decrease in the flavonoid content in pollen grains of several citrus species [47]. Thus, changes in flavonoid content could provide information about the effects and intensity of stress. In addition, flavonoids play a critical role, especially in the UV-B stress response; they could reduce the penetration of incident UV radiation or act as quenchers of reactive oxygen species [24]. Therefore, pollen grains accumulate flavonoids to protect them from UV-B damage and preserve their viability after anthesis [24].

According to the results, there are no significant differences in the scavenging activity of DPPH radicals in the three experimental groups with the highest harmful effects on pollen grains. However, phenolic compounds decreased significantly only after 3 h of treatment at 10 cm and after 3 h of treatment at 20 cm. In addition, UV-B radiation significantly reduced the flavonoid content of all experimental groups compared to the control. All of these results indicate that UV-B radiation affected the antioxidant system. According to the literature, conditions of intense stress could deactivate antioxidant enzymes, at the same time upregulating the biosynthesis of flavonoids. That is why the activity of flavonoids is considered a “secondary” antioxidant system, which is activated due to the depletion of antioxidant enzyme activity [49]. Contrary to the literature, the unchanged radical scavenging activity and the decrease in phenolic compounds could hypothetically be related to the short (3 h) exposure of pollen to UV radiation. Consequently, we can assume that UV have first a destructive effect on phenolic compounds. Given that phenolic compounds are involved in pollen development, pollination, pollen germination, and pollen tube growth [48], the sharp decrease in phenolic content could explain the resulting decrease in pollen germination and tube length after three hours at 10 cm. In addition, flavonoids are known to promote pollen tube elongation [50]; pollen germination and tube length also increase when flavonoids are added to the germination medium of *Nicotiana tabacum* [51]. In view of this, the decrease in flavonoid content along with the reduction in viability, germination and tube length could explain the detrimental effect of UV-B radiation on pollen. Pollen grains are likely not capable of increasing flavonoid content or require a longer exposure time, so a few hours may not be enough to increase flavonoid content. Alternatively, the genetic pathway for flavonoid synthesis is deactivated in pollen after it has been released from anthers; in fact, it is known that pollen grains accumulate flavonoids for protection from UV-B and to preserve their viability after anthesis [24].

Pollen grains, like other plant tissues and cells, produce UACs as a repair and defense mechanism against UV-B exposure [52]. The content and variation of UACs after exposure to UV-B differ between pollen grains of different species and even between pollen grains of different genotypes [53]. Therefore, the content of UACs is a useful tool to monitor the effect of UV-B radiation on pollen grains [37]. Indeed, UAC production in pollen grains has been shown to increase as a direct response to increased UV-B exposure [53,54]. Day and Demchik [18] reported a strong increase in UACs after UV-B treatment in *Brassica napus* pollen grains. Santos et al. [40] also showed a significant increase in UACs in *Zea mays* pollen after UV-B exposure. It should be emphasized that not all results are in line with the increase in UACs after UV B treatment. In fact, Musil [55] reported that UACs were not significantly affected by UV-B in pollen grains of *Setiecio elegatis*, *Petzia suffrtiticosa*, *Ursitiia attthetnoidc*, *Poiret situiata*, *Ditnorphotheca situiata*, *Ixia viridiflora*, *Gladiohis catneus*, *Geissorhiza tadians* and *Bahiatia rtthrocyatica*. In this work we found that UACs of the pollen fractions decreased after UV-B treatment. However, the pattern is different between the three experimental cases. Cytoplasmic UACs decrease sharply after 2 h at 20 cm, while cell-wall-bound UACs show a linear decrease that is inversely proportional to the time/distance of treatment. The UACs of the sporopollenin-derived fraction behave differently since they increase after 2 h of treatment at 20 cm, but then decrease. Although we ignore the relative contribution of each UAC fraction to UV-B tolerance, we can assume that sporopollenin is more active than other fractions due to the persistence of UACs after UV-B treatment. From a general point of view, we can say that the content of UACs is reduced in hazelnut pollen after exposure to UV-B radiation. This contrasts with what is reported in the literature and suggests that the UACs in hazelnut pollen may not be as effective as in other species for protection against UV-B radiation [33]. The decrease in UAC content may be one of the reasons for the decrease in pollen viability, germination, and tube length.

Sequential extraction of UV-B absorbent compounds showed that the absorbance of the cytoplasmic fraction was the highest, with intermediate absorbance by the sporopollenin-derived fraction, while the cell-wall-bound fraction gave the least absorbance. This finding is comparable to data obtained in other species where the cell wall fraction makes the least contribution to UV-B absorbance [33,37]. This is not surprising since the cell wall probably contains a lower number of UACs than the cytoplasm and sporopollenin fraction. Contrary to our findings, Rozema et al. [33] found that the sporopollenin-derived fraction had the highest absorbance, while the cytoplasmic fraction showed intermediate absorbance in *Helleborus foetidus* naturally exposed to UV-B. This difference may also be due to the variation (quantities and types of phenolic and other compounds) of chemical constituents of sporopollenin between different species [56,57]. However, another study by Rozema et al. [33] in *Betula pendula* (belonging to the same family as our study material) showed that the cytoplasmic fraction exhibits the highest absorbance while the sporopollenin-derived fraction is characterized by intermediate absorbance. Sporopollenin can absorb about 80% of the UV-B radiation to which pollen grains are exposed [18]. This suggests that sporopollenin is the most protective part of pollen grains against UV-B radiation. However, UACs of pollen grains originate in the cytoplasm, and this may explain the high absorbance of the cytoplasmic fraction in our results [58]. In addition, the high absorbance of cytoplasmic UACs is very important to protect pollen grains against 20% UV-B radiation that penetrates the pollen cytoplasm and is potentially capable of damaging the generative cell [18]. Speculatively, we can assume that hazelnut pollen synthesizes UACs that remain more concentrated in the cytoplasm, and only a few of them reach the cell wall and then sporopollenin.

## 4. Materials and Methods

### 4.1. Plant Material and Application of UV-B Treatment

Pollen materials were collected from Akçakoca/Düzce (Turkey) in February 2022 in a hazel orchard located in West Black Sea Region, Akçakoca/Düzce (Turkey) (41°05′40.2″ N, 31°15′09.1″ E). After dehydration in silica gel overnight, pollen grains were stored at −20 °C. UV-B radiation was provided by a PL-S 9W UV-B lamp (Philips) with narrow waveband between 290 and 315 nm. An acetate film was placed in front of the lamp to filter out possible UV-C radiation. The pollen grains were placed under UV-B lamps at distances of 10, 20, 30, and 40 cm and exposed to UV-B for 1, 2 and 3 h. No treatment was applied to the pollen of the control group. Before each application, the homogeneity of UV-B radiation at different distances was measured with a broadband spectroradiometer [23].

### 4.2. Pollen Viability

To observe the effect of UV-B radiation on pollen viability, Fluorescein Diacetate (FDA)/Propidium Iodide (PI) stain was prepared [59]. Briefly, 2 mg FDA was dissolved in 1 mL acetone and diluted by 12% sucrose drop by drop until turning milky. The solution was mixed with the PI solution prepared by the dissolution of 1 mg PI in 1 mL PBS. Pollen grains were mixed with 12% sucrose, and FDA/PI stain was added to the pollen solution in equal volume. After incubation for 10 min in dark, excess of the stain was removed by washing 3 times with 12% sucrose solution at 7000 rpm. Pellets were resuspended with 12% sucrose and observed under fluorescence microscope at 500–650 nm (Olympus BX-51). Fluorescently green-labelled pollen grains were considered viable, while red grains were not viable. Experiments were conducted in triplicates and pollen viability percentages were scored by considering 300 pollen grains for each individual treatment using ImageJ software.

### 4.3. In Vitro Pollen Germination and Tube Length

To evaluate the effect of UV-B radiation on pollen germination and tube length, pollen grains were germinated at 20 °C with 50% relative humidity for 24 h, according to previous studies [7,59]. The medium BK with 12% sucrose was used as a germination medium [60]. Pollen grains were considered as germinated when the tube length was higher than the pollen diameter. Experiments were conducted in triplicates. Pollen germination rates were measured by counting 300 pollen grains and tube lengths were measured considering about 150 germinated pollen grains using an optical microscope (Olympus BX-51) and ImageJ software.

### 4.4. Determination of Cumulative Stress Response Index

To identify the type of treatment that caused the most sensitive effects on pollen grains, the CSRI value of all groups was calculated using the equation of Dai et al. [61] with modifications. The CSRI was determined from the viability rate (VR), germination rate (GR) and tube length (TL) of the control (c) and treated (t) groups using the following equation:

(1) $$\mathrm{CSRI}=\left(\frac{\mathrm{VRt}-\mathrm{VRc}}{\mathrm{VRc}}+\frac{\mathrm{GRt}-\mathrm{GRc}}{\mathrm{GRc}}+\frac{\mathrm{TLt}-\mathrm{TLc}}{\mathrm{TLc}}\right)\times 100$$

Standard deviations (SD) of CSRI values were calculated, and UV-B treatment groups were sorted according to the following equation as sensitive, average, or tolerant:

Sensitive <minimum CSRI + 1 SD < Intermediate < minimum CSRI + 2 SD < Tolerant < minimum CSRI + 3 SD

As we aimed to reveal the damaging effects of UV on pollen grains, three treatment groups showing the most acute effects were selected using the CSRI value. Based on this classification, only the experimental groups causing sensitivity in pollen were studied in further experiments.

### 4.5. Determination of Total Antioxidant Activity, Total Phenolic and Flavonoid Contents

To study the effect of UV-B radiation on total antioxidant activity, total phenolic and flavonoid content, 0.05 g of pollen grains were extracted in 3 mL of methanol by ultrasonication for 30 min. The solution was centrifuged at 10,000× *g* for 15 min, and the supernatants were used for assay of total antioxidant activity, total phenolic compounds, and total flavonoid compounds [62]. To determine the total antioxidant activity, the free radical scavenging activities of pollen extracts were determined using DPPH (1,1-Diphenyl-2-picrylhydrazyl) radical [63]. A total of 50 µL of extracts was mixed with 100 µL of DPPH solution. After incubation in the dark for 30 min, absorbance was measured at 517 nm spectrophotometrically. Methanol was used as a blank and L-ascorbic acid (vitamin C) as a positive control. Experiments were conducted in triplicates. The %DPPH radical scavenging activity was calculated by the following formula:

%DPPH radical scavenging activity = [(A blank − A treatment)/A blank] × 100

Total phenolic contents were determined by the method of Clarke et al. [64]. In total, 40 µL of pollen extracts, 100 µL of Folin–Ciocalteu (diluted 1:10 in distilled water) and 75 µL of 7.5 percent Na_2_CO_3_ were added to 96-well microplates After a 1 h incubation, absorbance was measured at 750 nm spectrophotometrically. Experiments were conducted in triplicates and methanol was used as a blank. The standard graph was prepared using gallic acid at a stock concentration of 1 mg/mL, and phenolic contents were tabulated according to gallic acid equivalence. Total flavonoid content was determined according to the method of Yang et al. [65]. In total, 75 µL of pollen extracts, 75 µL of 2% AlCl_3_ solution and 75 µL of sodium citrate solution were added to 96-well microplates. After a 15-minute incubation, absorbance was measured at 435 nm spectrophotometrically. Experiments were conducted in triplicates and methanol was used as a blank. The standard graph was prepared using rutin at a stock concentration of 1 mg/mL, and flavonoid contents were plotted against rutin equivalence.

### 4.6. Sequential Extraction of UACs in Pollen Grains and Fluorescence Microscope Analysis

For the analysis of UV-B absorbance from different pollen fractions, the UACs of pollen grains were extracted sequentially. To confirm the efficiency of the extraction process, we examined pollen grains under a fluorescence microscope before and after extraction procedures. Samples were mounted with dH_2_O and 300 pollen grains from each group were observed under a fluorescence microscope with the following emission filters: 416–477 nm for the green channel, 498–550 nm for the red channel and 572–620 nm for the blue channel [66]. In addition, 300 pollen grains from each group were examined under the light microscope. For UV-B absorbance analysis of UACs from different pollen fractions, 0.05 mg of pollen was mixed with 10 mL of solution containing MeOH–H_2_O–HCl (79:20:1 *v***/***v*) and kept in a water bath (90 °C) for 2 h. After centrifugation at 3500 rpm for 15 min, the supernatant was carefully removed and used as the source of UACs from the soluble (cytoplasmic) fraction; the relative absorbance of the supernatant was measured with Beckman Coulter spectrophotometer (DU 730) at 300 nm [33,67]. The pellet was air-dried and mixed with 10 mL of 2 M NaOH. The solutions were sonicated for 5 min and then incubated at room temperature for 1 h under stirring. After centrifugation at 3500 rpm for 15 min, 37% HCl (220 µL) was added to the supernatants, thus adjusting the pH to 1.0; these solutions were used for cell-wall-bound fractions, and the relative absorbance of the supernatant was measured as described previously [33,67]. The pellet was mixed with 1 mL of solution containing 95% H_2_SO_4_ and acetic acid anhydride (1:9, *v*/*v*). The solutions were stored in a boiling water bath (90 °C) for 1 h and constantly mixed. After cooling, the solutions were centrifuged at 3500 rpm for 15 min. Subsequently, the pellets were washed twice with acetic acid, and then washed three times with distilled water. The brown residue obtained after this step was resuspended with 1 mL glycerol and the mixture used as the sporopollenin-derived fraction; the relative absorbance of the supernatant was measured as described above [33,68]. All the experiments were conducted in triplicates.

### 4.7. Statistical Analysis

Statistical analyses were performed by SPSS 16.0 software and data were subjected to one-way analysis of variance (ANOVA) with a threshold *p* value of 0.05.

## 5. Conclusions

Exposure to UV-B rays for different times and distances damages pollen to different levels. The damage is clearly demonstrated by the reduction in the content of antioxidant molecules such as phenols and flavonoids. Although the relative protection of different pollen fractions is not clear, cytoplasmic UACs are more abundant but more sensitive to UV-B exposure. As a result, the negative effects of UV-B on antioxidant molecules and UACs are accompanied by a decrease in pollen viability, germination rate and tube length. This manuscript provides fundamental information on the effects of UV-B radiation on hazelnut pollen. Although further experiments are clearly needed to elucidate the molecular targets of UV-B radiation and the different responses that pollen puts in place, these data clearly show that UV-B radiation is harmful to economically important crop species such as hazelnut. Again, this underlines the need to deepen studies on this abiotic stress in relation to plants of agricultural interest. We are also confident that the results will highlight similar mechanisms in other important crop species, which base their economic importance on fertilization.

## Figures and Tables

**Table 1:** CSRI values of treatment groups.

Treatment Times				
		1 h	2 h	3 h
**Treatment** **distances**	**10 cm**	−39,941	−89,516	−122,685
	**20 cm**	−42,664	−146,282	−106,176
	**30 cm**	−28,603	−46,672	−94,886
	**40 cm**	−11,915	−42,361	−67,687
Sensitive < −104, 7 < Intermediate < −63, 2 < Tolerant < −21, 7				

## Data Availability

Not applicable.
